# Supplementary material for: How positive deviants helped in fighting the early phase of COVID-19 pandemic? A qualitative study exploring the roles of frontline health workers in Nepal
Source: PLOS Glob Public Health. 2023 Mar 8;3(3):e0000671. doi: 10.1371/journal.pgph.0000671 (PMC10021390; doi:10.1371/journal.pgph.0000671)
Supplement: S1 Table — (DOCX) [file pgph.0000671.s001.docx]

**S1 Table Interview guide**

| 1 | **Introduction**   - Tell us about yourself: your education background, current place of work, years of working, - Tell us about your roles and responsibilities in your current place of work |
| --- | --- |
| 2 | **General working experience in health facility**   - How does your normal day at work look like? What are your specific roles during normal days? - How is the patient load in general?   (*Probing: General patient and COVID patient*)   - What kinds of patients do you manage?   (*Probing: two types of patients general and critically ill patients, for peripheral area more general patients with less or no respiratory symptoms, in central with ICU facilities more critically ill patients.)*   - How are your working hours like?   (*Probing: duty hours, working with specific group of team, patient care etc*) |
| 3 | **Work life during COVID-19**   - How has COVID-19 changed your daily schedule? - [workplace, families] *(probing: physical distancing from family members, self-quarantine, not mixing with family and friends)* - How is the support from your family, your colleagues/staff? *(probing: encouraging / discouraging /love/hate/respect)* - How is the support from locals, local govt, provincial and central govt*?(probing: support can be logistics, administrative like perks, leaves, insurances, moral support)* - What kind of precautions are you taking? *(probing: personal, and at the hospital settings?)* |
| 4 | - **Challenges of COVID 19** - What are the current challenges your facility is facing*? (probing: in terms of logistics, staffs, remunerations ?)* - What are the challenges you as a doctor is facing right now/during COVID-19? *(probing: personal and the working site, stigma, discrimination)* - How is the coordination with local government/ provincial and central government? *(probing: in terms of logistics, remunerations, referral, trainings)* |
| 5 | - **Positive deviance during COVID-19** - Have you or your colleagues tried to bring positive changes in your work place or your community? If yes, could you please share your experiences? - Have you or your colleagues found new innovations to tackle the crisis in your work place or your community? If yes, could you please share your experiences? (*in terms of management, equipment’s, as a team)* - What are the positive lessons to other front line health workers that they can learn from your / your team's working experiences?   *(probing: in terms of management, logistics)*   - What are the positive lesson you have learned from other front line health workers from the public health emergency / pandemic?? *(probing: self and work site, collective work, team work, mutual trust and cooperation)* - How things can improve in the coming days for the betterment of society as a whole? *(probing: ideas, awareness, team work, use of locally available resources )* |
| 6 | **Any last message or suggestion to the concerned stakeholders of COVID 19**  (such as authorities in health workforce, government, community leaders etc?) |
| 7 | **Any more stories you’d like to share?** |
|  |  |

**Thank you for your valuable time!**
