# Supplementary material for: How positive deviants helped in fighting the early phase of COVID-19 pandemic? A qualitative study exploring the roles of frontline health workers in Nepal
Source: PLOS Glob Public Health. 2023 Mar 8;3(3):e0000671. doi: 10.1371/journal.pgph.0000671 (PMC10021390; doi:10.1371/journal.pgph.0000671)
Supplement: Attachment — Submitted filename: Responses to reviewers.docx [file pgph.0000671.s003.docx]

**Responses to the editor and reviewers**

Dear Editor and reviewers,

Thank you for your constructive comments. We have tried our best to address all your comments and revised the manuscript as suggested. Below please find the point-by-point responses to each comment. We look forward to your positive response.

| **Editor’s comments** | **Responses** |
| --- | --- |
| Journal Requirements:  1. In the online submission form, you indicated that "Data will be made available upon reasonable request as the data is not entirely anonymous.". All PLOS journals now require all data underlying the findings described in their manuscript to be freely available to other researchers, either 1. In a public repository, 2. Within the manuscript itself, or 3. Uploaded as supplementary information.  This policy applies to all data except where public deposition would breach compliance with the protocol approved by your research ethics board. If your data cannot be made publicly available for ethical or legal reasons (e.g., public availability would compromise patient privacy), please explain your reasons by return email and your exemption request will be escalated to the editor for approval. Your exemption request will be handled independently and will not hold up the peer review process, but will need to be resolved should your manuscript be accepted for publication. One of the Editorial team will then be in touch if there are any issues. | Thank you for the suggestion  We have now mentioned that  All underlying data is available within the manuscript itself and in supplementary files. |
| 2. We have noticed that you have uploaded Supporting Information files, but you have not included a list of legends. Please add a full list of legends for your Supporting Information files after the references list. | The full list of legends for supporting information has been added after the references list. |
| **Reviewer #1:** |  |
| Dear Authors, it was a pleasure reading your manuscript. Congratulations on your findings and using the innovative method to look at the perspectives of frontline HCWs from a different angle. Although I am not a core qualitative researcher nor a social scientist, who could have given more detailed feedback, as a general medical/public health researcher, I have the following comments for your consideration: | Thank you for your encouraging words and constructive comments. We have tried our best to address the comments and revise our manuscript. |
| Abstract: Please elaborate results section. Introduction and method section could be reduced. Mention key sub themes, important traits, etc | Thank you for the suggestion. We have now reduced the results section and elaborated the results section mentioning the key subthemes in the abstract section of the revised manuscript. |
| Methods/Results:  - Under types hospital, readers would like to see the difference in findings between public and private healthcare facilities. Out of the selected health facilities, how many of them were recognized as COVID-19 designated HCFs by the government? Was there any difference in findings between designated and non-designated HCFs? | We have elaborated on the differences and similarities in the experiences among the health workers from different types of health facilities in the results section as well as in discussion section.  We have also clarified that at the time of the interview, only the district, provincial, and central hospitals were designated as COVID-19 hospitals. The primary health centers were not designated as treatment centers for COVID-19, however, they were responsible for managing the quarantine and isolation centers within their communities. This has been elaborated in the results section as well. |
| Results:  - Table 2: Please write the legend in full. What do these codes, categories and themes reflect or relate to? COVID-19 response in resource-poor healthcare settings? | We have added the legends in full as suggested.  Table 2 has not been moved to the results section and is labeled as Table 1 based on Reviewer 5’s suggestion. |
| - It would be better to give numbers to sub themes (for e.g., 1.1 personal challenges, 1.2 professional challenges) for easier reading and understanding. | Thank you for the suggestion. We have included numbering for the subthemes as suggested in Table 1 and results section. |
| Discussion:  - It would be helpful to discuss the differences of findings (motivation, challenges/experiences, recommendations) across health profession/cadres, types of HCFs (public vs private), and rural/urban settings. | We have now elaborated the discussions on differences in findings in the discussion section. |
| General: Although it is a qualitative study conducted among 17 participants, readers would like to see the deviation of study findings across different cadres/profession, types of hospital, rural/urban setting, etc. measured in proportion or percentage. Please review statistical aspects. | The table on characteristics of the participants has been revised as suggested. (Table 2) |
| **Reviewer #2: Comments:** |  |
| Major comments: |  |
| 1. No clear mention of how those 17 participants were selected for the interview | It has been elaborated in the Materials and methods section under the selection of the participants. |
| 2. Pertinent and relevant discussion of the author's research finding is mentioned in the discussion; however, it is recommended to discuss more the psychological impact among health care workers from Nepal in addition to the global impact authors have mentioned in their second paragraph of discussion, including findings from some pertinent literature <https://pubmed.ncbi.nlm.nih.gov/34345537/>, <https://pubmed.ncbi.nlm.nih.gov/33566863/>, etc. | Thank you for the feedback. We have included relevant findings regarding healthcare workers' mental health during COVID-19, citing local literature in the discussion section (paragraph 2). |
| **Minor comments:** |  |
| 1. The introduction is too lengthy and contains very general information, which may not be necessary for the reader of scientific journal articles. Authors may keep some of that information in the discussion section instead | We have now removed the general information and tried to keep it more concise. |
| 2. Unsure about the actual date versus typo error? "The interviews were conducted between 8 December November 2020 and 16 March 2021 and there were no repeat interviews." | Thank you for pointing out this typing error. We have corrected it in the methods section under data collection.  The interviews were conducted between 8 November 2020 and 16 March 2021 |
| 3. Poof reading and minor edits of English and language-related issues are recommended | We have thoroughly attempted to eliminate grammatical errors in the revised manuscript. We have also tried to break down complex sentences into simpler forms. We have also attempted to maintain consistency in parts of speech, voice, tense, and overall sentence structure. |
| **Reviewer #3:** |  |
| • Line 56-57: Reference required  • Line 58-59: Reference required  • Line 74-87: Reference required  • Line 77-81: Reference required | Thank you for pointing this out. We have added relevant citations to these texts. |
| 2 Materials and Methods |  |
| a) Steps of positive deviance approach:  • Please paraphrase and shorten the paragraphs. It is very confusing at the moment. The description/steps of the  positive deviance initiative should be at the beginning to introduce the concept and the differences followed by  it. | We have revised and shortened the paragraphs to provide more clarity.  We have now revised the stages of the positive approach that is more applicable within the health care setting. |
| b) Data Collection: |  |
| • Please elaborate how the privacy and confidentiality was maintained. | We have elaborated it under data collection. |
| • Please write the exact data collection period. Is it November or December 2020? | Thank you for pointing out this typing error. We have corrected it in the methods section under data collection.  The interviews were conducted between 8 November 2020 and 16 March 2021 |
| • Did you obtain a written informed consent or a verbal informed consent? Specify. | We obtained the video-recorded informed consent before the interview where the participants declared that they had read through the information sheet and were willing to participate in the study. This has been elaborated under the ethical considerations section. |
| 3 Results |  |
| • The interpretation in regards to the themes and subthemes are inadequate. Some subthemes directly consist of  the quotation without any interpretation and this is not correct. Quotations are there for supporting your  interpretation not a replacement for it. | Thank you for the suggestion. We have now provided interpretation for all the subthemes in the results section. |
| • The subtheme- Finding solutions within the theme overcoming Challenges more or less corresponds to  innovative solutions. Why not you name this theme as Challenges and then only present personal and  professional challenges. | Thank you for the suggestion. We have now moved “Finding solutions” to the “Innovative solutions” theme  We have also renamed the first theme as Challenges. |
| • May be you can merge subthemes Practice based innovations and technological innovations/Practices . | We have now merged the two subthemes as suggested. |
| • You have not written anything on the subthemes: Lessons from organization and lessons from government. | We now added the interpretation for the theme of positive lessons. |
| Please check.  • Line 297-301: What does 5 PPE mean? Is it the PPE set or a particular PPE? | We have provided the full terminology as Personal protective equipment set (PPE) and used the acronym PPE after that. By 5 PPE the participants meant PPE sets. |
| Overall: Please cut down the use of the article “The” | We have attempted to ensure the appropriate use of the article. |
| Use gender neutral terms as far as possible. E.g Replace manpower with human resource | We have replaced “manpower” with “human resources”  We have also tried our best to use gender-neutral terms throughout the text. |
| Check the grammar in the whole manuscript. | We have run a thorough grammar check for the entire document. |
| Use active voice rather than passive specifically in the methods section | We have switched the passive voice to active voice in the methods section. |
| Reviewer #4 |  |
| The study is usefult to gain insights about positve deviants that front health workers experienced during the early phase of COVID-19 in Nepal. The authors could benefit from the following suggestions: | Thank you for your positive comments and valuable suggestions. |
| 1. Methods, Line 158-159, There seem to be an error in reporting the duration of data collection. Please correct it  2. Methods, Line 161-164, Was the consent taken in written form or video-recorded during the interview? | Thank you for pointing out this typing error. We have corrected it in the methods section under data collection.  The interviews were conducted between 8 November 2020 and 16 March 2021  We obtained the video-recorded informed consent before the interview where the participants declared that they had read through the information sheet and were willing to participate in the study. This has been elaborated under the ethical considerations section. |
| 3. Results, Line 180-182, Can the number of participants be disaggregated by gender and also by province? Was there any specific reason for choosing more participants from Mountain than plain?  I was particularly concerned about why health workers like laboratory personnel were not included in the study. | We have revised the table for the characteristics of the participants. The participants are now also disaggregated by gender, age groups, types of health facilities, and location (urban vs rural).  We acknowledge that there is an underrepresentation of health workers like laboratory personnel and other paramedics. We have elaborated it in the limitations section |
| 4. Results, Line 189, Code of Theme 5 (Recommendations) seems to be missing. | We have revised the themes and the theme has now been merged with the theme of lessons. |
| 5. Discussion, Line 642-657, This paragraph is more about explaining what intrinsic and extrinsic motivation are. I would like to suggest authors to relate to the study findings, describe it or compare it with other settings and draw implications from it. | We have now revised the paragraphs discussing motivation based on the findings and have compared it with other studies and drawn implications from it. (Discussion- paragraphs 7 and 8) |
| 6. Please correct some typos such as 'FInding solutions' (Line 189) | Thank you for pointing this out. The typing error has been corrected. |
| Reviewer #5: |  |
| This is a good and relevant study and my hearty congratulations to the authors for working on an interesting topic. I feel that the study is interesting, but the paper needs more details and revisions for it to be well interpreted by an external reader. Sharing some suggestions below.  I know this list might look a bit overwhelming at first (my very sincere apologies) but hope it is helpful in moving forward. | Thank you for your thorough review and constructive comments. |
| Introduction |  |
| • The Positive Deviance approach needs to be defined clearly, using references in the first sentence.  Sentence 42-43 may need to be tweaked since PD as a practical strategy enables social and behavioral changes through looking at outliers (does not address social and behavioural changes though). I think in this paper, PD is used to identify ‘champions of change’, outliers who succeeded despites odds during a very challenging situation.  This paper has a good overview of various concepts of definitions of PD-Herington MJ, van de Fliert E. Positive Deviance in theory and practice: a conceptual review. Deviant Behavior. 2018;39(5):664–78. | Thank you for the suggestion. We have revised the sentence as suggested and added the suggested citation in the second sentence of the first paragraph in the introduction section.  . |
| • Line 52- few more paper references can be used rather than only one. Particularly papers that have used PD approaches to model frontline health worker behaviours previously (rather than those that looked at community behaviours) | Thank you for the suggestion. We have tried to add more references related to the use of PD in health care settings. We also added the citations of the studies that were used to explore positive deviance among frontline health workers in the introduction section. (Paragraphs 4 and 5) |
| • Line 53- tweak. PD approaches can’t be used to address the pandemic. Restate-PD approaches haven’t been used to study frontline workers in the context of COVID-19. I like lines 82-83 that convey this point better. | We have paraphrased the sentence as suggested. (First line in paragraph 4) |
| • Lines 81-82 have good arguments for the selection of positive deviants. | Thank you for your encouraging comments. |
| • Lines 87-88: it could be interpreted as the frontline workers interviewed were diagnosed with COVID-19. Aim of the study - suggestion- explore the experiences of positively deviant frontline workers during early days of the pandemic | We have revised the sentence as suggested to avoid confusion in the last paragraph of the introduction section. |
| Methods |  |
| • Identification and selection of participants has been clearly written, data collection processes are also very well defined. | Thank you. |
| • It would be good to have a small box/table on the themes covered during the interviews. | We have now included the interview guide as a supplement table to provide more clarity on the themes covered during the interviews. |
| • Data analysis lines 172-176 are a little confusing for an external reader. Was the coding tree created before the reading of the transcripts? (from the interview guide) | We have restructured the data analysis section to provide more clarity |
| • Table 2 with codes can come into the analysis section as it usual practice in qualitative papers. | As suggested, we have now moved the table from the results section to the data analysis section under the part explaining coding to provide more clarity. The table is now labeled as Table 1. |
| • I am a little confused about the methods followed. Lines 111 -115 state that the steps defined by the positive deviance initiative were followed for the research. But the findings are not reported in terms of these steps. I see step 2 (identification of positive deviants) and a bit of step 3(discovering successful behaviours). I don’t see the other steps. | We have revised this section and used a more suitable reference for stages of the positive deviance approach and we have highlighted that we focused on the initial two stages of the positive deviance approach. |
| • Table 1 could have age, gender and other details- as usual demographic tables in qualitative papers. | We have revised the table and added more demographic information. The table is now labeled as Table 2. |
| Findings |  |
| • This section may need a lot of rewriting. It has many quotes and very rich, beautiful quotes- but these need to get tied into a narrative better. It is important to present quotes from the ground, but much of qualitative writing is about interpreting and weaving a story around these quotes. Some of this story is in the discussion section. Overall, the section has many quotes with very little reflection from the authors side on what these quotes mean or how can these fit together to tell a story. Hence, I would suggest major rewriting. | Thank you for the suggestion. We have made major revisions to this section. |
| • Lines 195 onwards-too many details on the challenges faced. | We have now shortened the details on the challenges. |
| • Maybe theme 1 Overcoming challenges section on “Finding solutions” can be merged with theme 2 “innovative solutions”. These two themes seem very similar. | We have merged the findings solution subtheme with theme 2 on innovative solutions. |
| • The theme on innovative solutions (line 322) feels like the meat of the paper to me. The findings here are fantastic and grounded, and we must tell the world about these! | Thank you for your appreciation. |
| • I do not understand the last theme on lessons at all. These is no text given by the authors to go along with the quotes. Much of the lessons seem like a reiteration of the overcoming challenges section in a different way. | We have added the interpretation of this theme. |
| • One way of tying these themes together is as below (only a suggestion and there can be other ways). First, you can describe (briefly) some of the challenges faced by frontline workers. Then a long description of how positive deviants attempted to overcome these challenges and the lessons they learnt during this process. This is the meat of the paper and used reflections/thoughts/ data displays like tables, quotes, even figures. Last, a section on what different factors motivated positive deviants to behave differently from others and find innovative solutions when others didn’t go so. | Thanks, we have restructured the themes as suggested. |
| • One data analysis book that might be useful is Miles, M.B., Huberman, A.M., 1992. Qualitative Data Analysis. | Thanks for the recommendation. The book was useful. |
| • Also it might help the authors to refer to how quotes are written in qualitative papers. Usually age and gender are used in quotes to give a better sense of the respondent profile. One cannot make out from these quotes the diversity in participants. | Age group and gender are now provided to the quotes |
| Discussion |  |
| • Has good referencing and the authors have attempted to synthesize lessons.  Some of the narrative here- example lines 636-640,  lines 658-666 all seem like findings rather than discussion.  In the discussion, I would be more interested in reflection about what these findings mean for future pandemics,  an action box for the way forward, etc. | We have made major revisions to the discussion and added more reflections and also compared with other studies on positive deviance.  We have also added the last paragraph where we discuss how the findings coils help the way forward. |
| Reviewer #6: |  |
| Thank you for the opportunity to review this paper – it was very interesting to read about the traits among positive deviants and their approaches used in managing the COVID-19 pandemic in low resource settings. | Thank you for your positive comments and constructive suggestions. |
| The following are my suggested revisions/questions to clarify in the report – |  |
| • In the background, I felt it would be useful to provide some examples of how positive deviance was used within the other public health programs mentioned (e.g. maternal and child health) | Thank you for the feedback. We have added examples demonstrating the successful implementation of the positive deviance approach in other public health programs such as maternal and child health. |
| • With regard to HCWs during the pandemic, it is mentioned that “Better knowledge and skills, better planning and management, etc. were some of the key qualities among these health workers which enabled them to perform better” – is there a reference for this? Did these findings also inform the conceptual framework/ interview guides used in the study? | We have tried to clarify the sentence. This sentence is in reference to what other studies suggested on the key qualities of frontline health workers. We have added the reference to this sentence.  The sentences were based on previous studies that were not related to positive deviance. |
| • In the methodology, it seems to me that the Positive Deviance Initiative is the approach that this study follows – I felt a clearer justification for using this approach was needed, and maybe the differences between the various approaches could be taken up in the discussion section. | We have revised this section to provide better clarity. We have now referred to the stages of positive deviance approach more applicable to the health care setting and have highlighted that we only focussed on the initial stages of positive deviance. |
| In the results, would it be possible to provide any further demographic details on the study participants (e.g. age, marital status, did they themselves have any medical conditions, etc.) and also how these could have affected the study findings | We have provided more demographic details and also elaborated the differences in the findings and discussions sections. |
| • I also felt further synthesizing of the findings presented in the quotes was needed for some of the themes and sub-themes reported (e.g. stigma, changing working patterns, psychosocial support for peers, etc.) | We have made major revisions to this section to provide more reflections and clarity. |
