# Supplementary material for: How positive deviants helped in fighting the early phase of COVID-19 pandemic? A qualitative study exploring the roles of frontline health workers in Nepal
Source: PLOS Glob Public Health. 2023 Mar 8;3(3):e0000671. doi: 10.1371/journal.pgph.0000671 (PMC10021390; doi:10.1371/journal.pgph.0000671)
Supplement: Attachment — Submitted filename: _Responses to reviewers- PD-2nd revision.docx [file pgph.0000671.s004.docx]

**Responses to the reviewers**

Dear Reviewers,

Thank you for your positive responses. We have tried our best to address all your comments and revised the manuscript as suggested. Below please find the point-by-point responses to each comment. We look forward to your positive response.

| **Reviewer #3** |  |
| --- | --- |
| The authors have addressed all the comments provided. There is no further comments from my side. | Thank you for your positive response. |
| **Reviewer #4:** |  |
| Authors have done a fair job addressing all the comments. Considering the knowledge contribution that the study brings to the COVID-19 pandemic and future outbreaks, I recommend the manuscript for publication | Thank you for your positive response. |
| **Reviewer #5:** |  |
| Thank you for your efforts in rewriting the article. I can understand that it is a lot of hard work bringing the manuscript to this phase and it is not easy to work with reviewer comments. My apologies for the same. | Thank you for your understanding. We have worked very hard to address all six reviewers’ comments in the earlier revision. We have also tried our best to address your comments in this revision. |
| I do still feel that some work is needed, particularly in synthesizing the data in the findings section. The quotes are still long. The discussion is also long winding and will benefit from some synthesis. I have given two sample papers below that might give a sense of how narratives can be written alongside quotes. | Thank you for your comments and for suggesting two papers to go through for our revision.  We have shortened the quotes from the findings. We have also shortened the discussion and tried to make it more comprehensive. |
| Abstract  Page 2- Line 19 Positive deviance is a concept that enables behavioral and social changes by learning 20 from the deviants who fare better than others under similar circumstances to find better solutions. Perhaps better to rephrase: Positive deviance is an approach wherein learnings from persons who fare better than their peers under similar circumstances are used to enable behavioural and social change. | We have rephrased the sentence as suggested in the revised manuscript.  (Abstract, page 2, Line 19-20) |
| Line 22 most socially and culturally contextual- fit into socio-cultural milieu, or fit the context well. | We have revised it as “fit into socio-cultural milieu”  (Abstract, page 2, Lines 21-22) |
| Introduction |  |
| • Definition of positive deviance tweaked | Thank you. |
| • The use of positive deviance versus deviants needs to be checked throughout the paper. | We have checked and corrected to retain the consistencies where applicable.  “Positive deviance” is used when describing the approach, and “positive deviant” when describing the people. |
| • Lines 57-77 can be condensed to make the point that such studies are widely used and useful | We have condensed it as suggested.  (Introduction, page 4, Lines 56-59) |
| • Line 78-79:rephrase. Given the usefulness of the positive deviance approach in understanding and enabling change in the health sector, the approach can be used in the context of COVID..etc ( References 14-16 are about positive deviants during COVID, so it might not be right to say that such studies have not been done at all) | We have rephrased it as suggested.  (Introduction, page 5, Lines 71-72) |
| • References 18 and 19 speak to Ebola and SARS in general, but do not convey the point that these did not affect Nepal. Need better references here. | We agree that these two references did not convey the point and therefore decided to remove the sentence and references. |
| Methods section  • Lines108-11 are very clear now.  • Liens 125 onwards is also very well written. The methods section is clear | Thank you |
| **Findings** |  |
| This section still needs work | We have tried our best to revise it. |
| • Quotes can be shortened and simplified. They are too long right now. | We have shortened and simplified the quotes. |
| • I feel that the connecting narrative still needs to be reflected upon and strengthened. For instance, there is only 2 lines of narrative on stigma, but three quotes. Another example is the section on managing resources- there is only one paragraph of narrative followed by 2 full pages of quotes. | We have expanded the narration for stigma and reduced the quotes to just 2.  For managing resources, we have reduced the quotes to 3 most illustrative ones from a total of 6 quotes. |
| • I would suggest to build a stronger expanded narrative that synthesizes the information you got from the quotes, but keep only 1 or 2s quote that is most illustrative of the points being made. | We have expanded the narrative where needed and reduced the number of quotes. |
| • Some sample papers below that use quotes and case-illustrations alongside the narrative (just to give ideas on how the narrative around the quotes can be strengthened). The narrative has to weave the ideas in the quotes together, and 1-2 quotes or case-stories may be used to illustrate key ideas.  [**https://bmjopen.bmj.com/content/bmjopen/12/6/e050363.full.pdf**](https://bmjopen.bmj.com/content/bmjopen/12/6/e050363.full.pdf)  [**https://www.ijhpm.com/article_3942.html**](https://www.ijhpm.com/article_3942.html) | Thank you for sharing these two papers. |
| Discussion- it would be nice to have one box that summarizes recommendations or learnings for policy from the study. | We have a paragraph on learnings for policy. To further clarify, we have added the subheading “Learnings for policy” to the paragraph.  (Discussion, page 40, Lines 685-691)  Presenting summaries in the boxes is not a format for this journal; therefore, we have presented the contents within the text. |
| The section can be shortened with clear suggestions or learnings. start the discussion with a clear summary of learnings (3-4 sentences). | We have shortened the discussion section.  As suggested, the first paragraph now includes the summary of key learnings.  (Discussion, page 34, Lines 561-566) |
| Usually the discussion section also has a small paragraph on the limitations of the study done, so it can be interpreted in context. | We have a paragraph on limitations. To clarify, we have added a subheading as “limitations” for the paragraph.  (Discussion, page 39, Lines 666-684) |
| **Reviewer #6:** |  |
| No further comments | Thank you for your positive response |
